# The Significance of Neutrophil Extracellular Traps in Colorectal Cancer and Beyond: From Bench to Bedside

**DOI:** 10.3389/fonc.2022.848594

**Published:** 2022-06-07

**Authors:** Dingchang Li, Jiakang Shao, Bo Cao, Ruiyang Zhao, Hanghang Li, Wenxing Gao, Peng Chen, Lujia Jin, Li Cao, Shuaifei Ji, Guanglong Dong

**Affiliations:** ^1^ Department of General Surgery, the First Medical Centre, Chinese PLA General Hospital, Beijing, China; ^2^ Medical School of Chinese PLA, Beijing, China

**Keywords:** neutrophil extracellular traps, colorectal cancer, metastasis, clinical application, tumor microenvironment

## Abstract

Neutrophil extracellular traps (NETs), products of neutrophil death when exposed to certain stimuli, were first proposed as a type of response to bacterial infection in infectious diseases. Since then, extensive studies have discovered its involvement in other non-infectious inflammatory diseases including thromboembolism, autoimmune diseases, and cancer. Colorectal cancer (CRC) is one of the most common malignancies in the world. NET formation is closely associated with tumorigenesis, progression, and metastasis in CRC. Therefore, the application of NETs in clinical practice as diagnostic biomarkers, therapeutic targets, and prognostic predictors has a promising prospect. In addition, therapeutics targeting NETs are significantly efficient in halting tumor progression in preclinical cancer models, which further indicates its potential clinical utility in cancer treatment. This review focuses on the stimuli of NETosis, its pro-tumorigenic activity, and prospective clinical utility primarily in but not limited to CRC.

## Introduction

Neutrophils, the most abundant white blood cells, play an important role in the immune system, especially for innate immunity (1). When exposed to exogenous pathogens, activated neutrophils can function in various ways, such as phagocytosis, cytokine release, and degranulation. Upon the activation of different signaling pathways, neutrophils may undergo different types of cell death, including apoptosis, necrosis, necroptosis, autophagy, pyroptosis, and NETosis (2). As a unique form of neutrophil programmed death, NETosis is characterized by the formation and release of NETs out of the cells. Neutrophil extracellular traps (NETs), mainly consisting of granule proteins and chromatin, were first described by Brinkmann et al. as a form of innate immune response to bacteria (3). Subsequently, several studies demonstrated the involvement of NETs in various non-infectious diseases, such as chronic inflammatory conditions, autoimmune disease, thrombosis, coronavirus disease 2019 (COVID-19) and malignancies (4–7). In addition, studies have reported that NETs mainly contribute to the progression of many types of tumors (7, 8). However, they can act as a double-edged sword, occasionally exerting anti-tumor effects (9).

Colorectal cancer (CRC) is the third most common cancer in the world, with approximately 1.3 million new cases and >600,000 deaths reported every year (10). CRC has a high probability of, and approximately half of the patients develop metastasis when diagnosed with CRC, which is also the main cause of death in patients (11). The currently available therapeutic strategies of CRC include surgical resection, chemotherapy, radiotherapy, and immunomodulatory therapy. However, approximately 40% of patients with CRC eventually have recurrence or metastasis, resulting in a 5-year survival rate of <15% (12, 13). Therefore, it is imperative to identify the exact mechanisms of CRC development and develop novel therapeutic strategies.

This review discusses stimuli that promote oncogenic NETosis, the structure of NETs and the involvement of NETs in non-neoplastic disease progression. Many studies have implicated that NETs can promote the progression of multiple tumors, including CRC, in different ways (14–16), indicating the importance of NETs in CRC and their potential value in clinical application. Therefore, in this review, we elucidated the impact of NETs mainly on CRC progression in terms of different stages and discussed the potential value of NETs in clinical application including diagnosis, therapeutic targeting, and prognostic prediction in cancer.

## Basis of NETs

### Stimuli of NETosis and Formation of NETs

**Figure 1:**
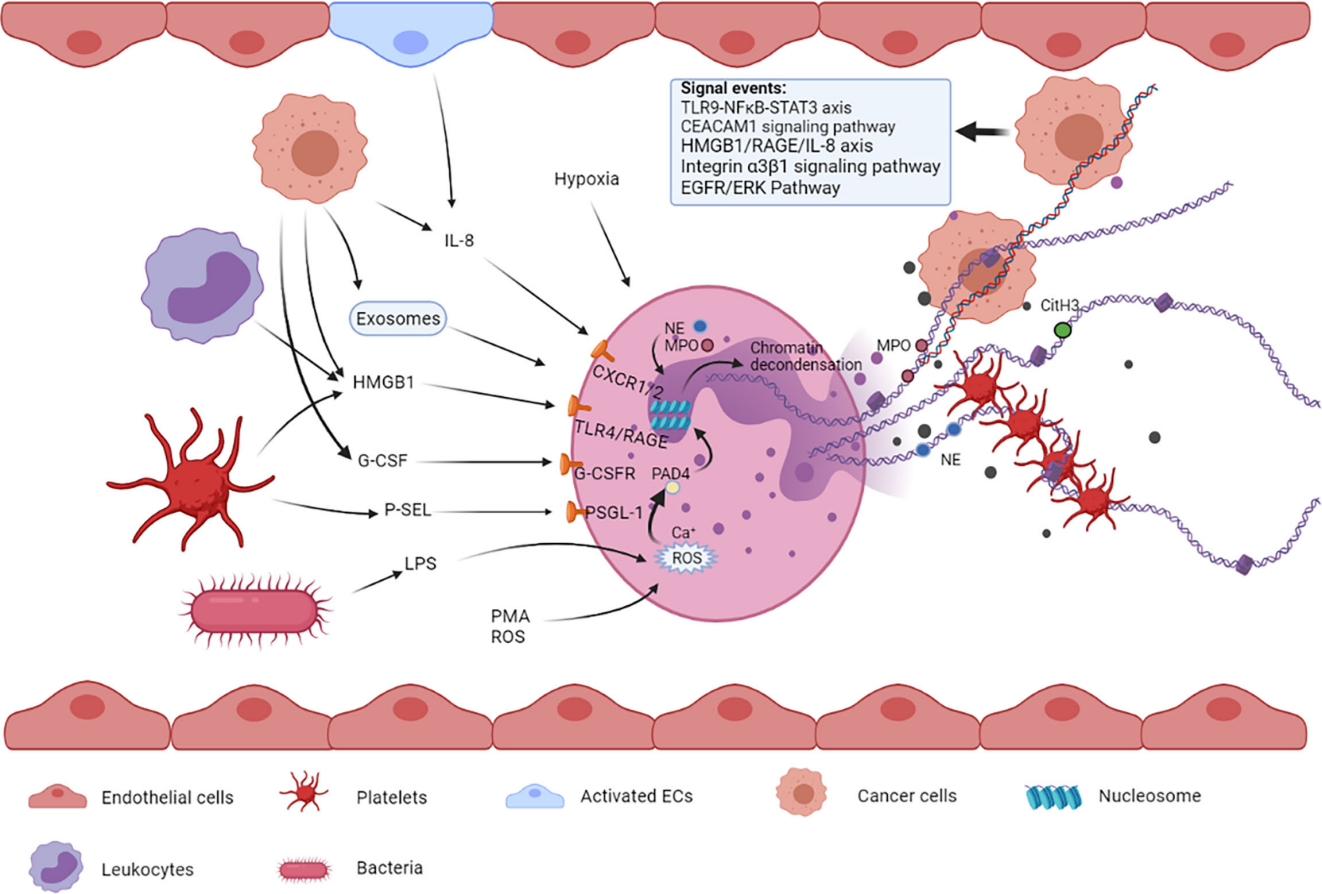
Multiple cells such as tumor cells, platelets, bacterial cells, and endothelial cells and their released factors are involved in NETosis. These factors from different types of cells can bind to their respective receptors on neutrophils, leading to NET formation. The released NETs are composed of a DNA skeleton and decorated granular and cytosolic proteins such as citH3, NE, and MPO. Thereafter, they can further activate and capture platelets, leading to venous thromboembolism. NETs can also entrap circulating tumor cells, promoting extravasation and metastasis.

NETosis is a process in which NETs are expelled out of neutrophils to the extracellular space. There are mainly two types of NETosis, namely, vital NETosis commonly occurring in infection and lytic NETosis often occurring in sterile injury (17, 18), which form NETs in two different ways, respectively. NETosis is also considered a form of cell death, different from apoptosis and necrosis, and is characterized by the extrusion of decondensed chromatin and protein contents to the extracellular space, forming NETs (19). A few important cellular events are involved in the process of NET formation, including the production of reactive oxygen species (ROS), migration of neutrophil elastase (NE) and myeloperoxidase (MPO) to the nucleus, histone citrullination, and chromatin decondensation (20). This series of events in neutrophils can be triggered under the influence of multiple cells and their paracrine components (**Figure 1**).

Lipopolysaccharide (LPS), one of the dominant components of Gram-negative bacterial cell wall, is a common inducer of NETosis. Many studies have shown that the formation of NETs is increased in patients with infection and have verified the effect of LPS on the formation of NETs to some extent (3, 21). In addition, LPS promotes NETosis *via* a ROS-dependent mechanism, and ROS production is closely associated with inflammation, cancer, and neutrophil modulation (22, 23).

Endothelial cells (ECs) can promote NETosis upon activation *via* exposure to oxidative stress in injury, inflammation, or some compounds such as phorbol 12-myristate 13-acetate (PMA) and thapsigargin (24, 25). Activated ECs can release inflammatory cytokines including interleukin-8 (IL-8) (26), which has been validated to partially participate in EC-mediated NET formation when co-cultured with neutrophils *in vitro* (25).

Activated platelets are also one of the contributors to NET formation (27). Upon activation, platelets can directly bind to neutrophils through multiple molecular interactions and subsequently stimulate NETosis (28). On the one hand, activated platelets can translocate P-selectin to their surface (29), where it can bind to the neutrophil surface receptor P-selectin glycoprotein ligand-1 (PSGL-1) and further strengthen their adhesion (30), eventually leading to the release of NETs. On the other hand, platelets can express toll-like receptor-4 (TLR4) and high-mobility group box 1 (HMGB1), which are important contributors to platelet-stimulated NETosis (21, 27). Simultaneously, the newly generated NETs activate platelets to achieve a prothrombotic state, thus forming a positive feedback loop between NETosis and coagulation (31).

Tumor cells can activate neutrophils to release NETs by promoting the expression and release of multiple pro-NETotic factors such as granulocyte colony-stimulating factor (G-CSF) (32), IL-8, and extracellular vesicles (EVs). Some studies have indicated that G-CSF can be secreted abundantly by cancer cells in either murine or human tumors and can activate neutrophils by binding to the G-CSF receptor on the surface of these cells (33, 34). Mélanie et al. showed that overexpression of G-CSF in patients with cancer can lead to an overabundance of neutrophils in the blood and increased sensitivity toward NET generation in a ROS-dependent manner (35). IL-8 is a common cytokine to recruit neutrophils to the sites of inflammation, which can be released by multiple cancer cells including glioblastoma, diffuse large B-cell lymphoma (DLBCL), bladder cancer, and CRC, and the released IL-8 can stimulate NETosis in human neutrophils *ex vivo* (7, 14, 36–38). In addition, the cytokine IL-1β is associated with NETs in some tumors (39, 40). Protein arginine deiminase 4 (PAD4) is an essential enzyme of NETosis, and its expression increases either in the blood or tumor tissues of patients with malignancies (41). High PAD4 levels provide enough enzyme for histone citrullination, which may be considered another potential way for tumor cells to promote NET formation. As a type of membrane-enclosed particles formed by membranes of the parent cell (42), EVs are considered one of the most crucial components that affect NETosis in cancer (43). According to recent studies, EVs promote NETosis mainly in two ways: First, through the effect of bioactive contents present in EVs, including ILs and G-CSF that induce NETosis (44); second, by inducing proinflammatory activity of neutrophils, for example, IL-8 secretion (45).

In addition to the abovementioned factors, microRNAs (miRNAs), a type of small non-coding RNAs that are involved in almost all physiological processes in the human body, modulate the process of NETosis (46, 47). Arroyo et al. revealed that miR-146a, a negative regulator of the inflammatory response, inhibited NETosis *in vivo*, and miR-146a knockout in neutrophils resulted in a higher level of citH3 and NETs relative to the wild-type neutrophils upon PMA stimulation (47). In addition, miR-155 promotes the generation of NETs by positively regulating the neutrophil expression of PAD4 (48).

Because NETs are prone to be induced in the inflammatory environment, which, in turn, can promote inflammation, we speculate that NETs are closely related to inflammation-associated factors. Theoretically, factors that promote inflammation can also promote NETosis. This understanding expands the range of influencing factors associated with NETs and provides a direction for further exploration of the inducers of NETs.

### Role of NETs in Disease Progression

In recent years, it has been discovered that NETs play an important role not only in the immune system but also in other pathologic processes such as thrombosis, autoimmune diseases, aseptic inflammation, metabolic disease, and the development of multiple tumors.

As a network in the circulatory system, NETs can capture and activate free platelets to promote thrombosis, especially during infection and inflammation (49). Many studies have validated the prothrombotic effect of NETs. For example, Fuchs et al. reported that NETs promoted fibrin deposition and thrombus formation, and blocking the production of NETs with DNA enzymes or PAD4 inhibitors reduced the incidence of deep vein thrombosis in animal models (50).

NETs released from neutrophils inevitably carry some intracellular proteins, that is, neutrophil-derived antigens. After these autoantigens are recognized by the immune system, they can drive the activation of autoreactive B cells to induce corresponding autoantibodies and result in autoimmune diseases (5). The association between NETs and autoimmune disease was firstly observed in patients with small-vessel vasculitis, whose serum was positive for anti-neutrophil cytoplasmic autoantibody (ANCA) against MPO and proteinase 3 in NETs (51). In some studies, NETs and autoantibodies have been detected in synovial fluid from patients with rheumatoid arthritis, and anti-citrulline protein antibody is the most significant of all autoantibodies (52). In addition, anti-ribonucleoprotein (anti-RNP) antibodies in patients with systemic lupus erythematosus (SLE) can induce NETosis, and SLE NETs activate plasmacytoid dendritic cells (PdCs) to produce high levels of IFN-α, which renders neutrophils more susceptible to NETosis upon stimulation of anti-RNP antibodies (53).

In addition, NETs are involved in some processes of aseptic inflammation. During the early inflammatory phase of atherosclerosis, cholesterol crystals can induce the generation of NETs, which, in turn, primes the transcriptional expression of IL-6 and IL-1β genes in macrophages and subsequently promotes the activation of T helper 17 cells, which enhance the recruitment of immune cells to atherosclerotic lesions (54). The pro-inflammatory effects of NETs have been demonstrated in the process of ischemia–reperfusion (IR) injury, during which neutrophils are recruited to the sinusoids of the ischemic liver lobes and release NETs in response to various stimuli, including the release of HMGB1 and histones from injured hepatocytes. In addition, inhibition of NET formation with PAD4 inhibitor or DNase I can protect hepatocytes and alleviate inflammation after liver IR injury, indicating the pathophysiological role of NETs in liver IR injury (55). Moreover, in mouse models of Alzheimer’s disease, NETs have been observed in areas with amyloid-β (Aβ) deposits. Depletion of neutrophils or blocking their trafficking process improves Alzheimer’s disease-like neuropathological features and cognitive performance in mice (56). As a nonspecific inflammatory bowel disease, ulcerative colitis (UC) has close relationship with NETs, which has been proven to be over-produced in inflamed colon of UC patients. The released NETs, in turn, significantly promoted IL-1β and TNF-α expression in mononuclear cells, leading to more NETs release (57). Meanwhile, the close relationship between UC and CRC has been acknowledged (58), which could be in part attributed to increased NETs in patients with UC.

Furthermore, NETs play a potential role in the pathogenesis of some metabolic diseases, such as obesity and diabetes mellitus (DM). Studies have shown that NET levels in patients with obesity are higher than those in the healthy population (59, 60), which is consistent with high levels of inflammatory cytokines in patients with obesity. Obesity is characterized by low-grade chronic inflammation, which underlies neutrophil activation and NET formation. Released NETs can, in turn, modulate inflammatory markers including IL-8, heat shock protein 90 (HSP90), and the E1 heat shock protein family (HSPE1), eventually contributing to metabolic profile alterations in obesity (60). Wong et al. reported that neutrophils derived from patients with DM were more susceptible to NETosis because of elevated PAD4 expression (61), which also means increased level of NETs in these patients compared to normal individuals. In addition, many studies have showed that obesity and DM were positively related to the incidence of CRC (14, 62, 63). Therefore, considering the role of NETs in CRC progression, high levels of NETs in patients with obesity and DM may be one of the reasons for obesity and DM being risk factors for CRC.

## NETs Promote Colorectal Cancer Development and Metastasis

**Figure 2:**
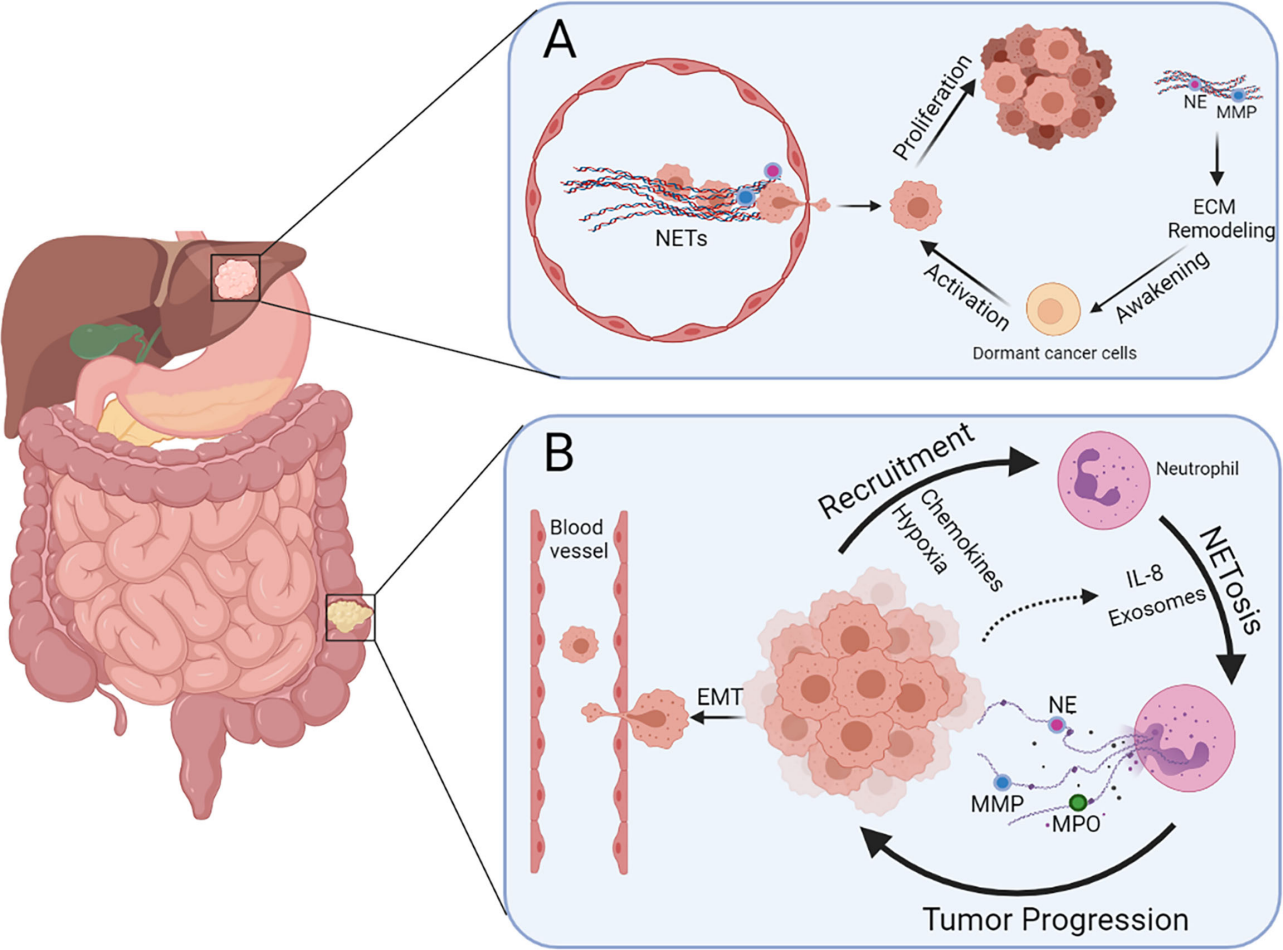
Multiple functions of NETs during colorectal cancer liver metastasis. **(A)** NETs present in blood vessels can entrap cancer cells and promote extravasation. Dormant cancer cells in the liver can be awakened *via* NET-induced ECM remodeling and hence promote metastasis and recurrence. **(B)** Neutrophils can be recruited by CRC cells because of the presence of chemokines and hypoxia in the tumor microenvironment, leading to more NETosis triggered by IL-8 and exosomes released by cancer cells. Cancer cells undergoing EMT are more prone to intravasate after stimulation of NETs.

The initiation and development of tumors are complex pathophysiological processes involving a series of genetic, biochemical, and molecular biological changes. To date, many studies have shown that NETs are involved in the development of various tumors, such as breast cancer, pancreatic cancer, gastric cancer (GC), and CRC. Moreover, systemic neutrophils derived from patients with CRC and age-matched healthy volunteers possess different vulnerabilities to NETosis, and the serum levels of NETs between them are also distinct (64). Therefore, to some extent, NETs have potential effects on CRC development. Furthermore, the role of NETs in the growth and metastasis of CRC and beyond in terms of three important stages of tumor progression, namely, the local microenvironment, circulatory system, and pre-metastatic microenvironment, is described below (**Figure 2**).

### NETs in the Tumor Microenvironment

NETs promote tumor growth in different ways in the tumor microenvironment. First, NETs can affect the state and infiltration of mesenchymal cells in the microenvironment to mediate tumor progression. In addition, NETs can promote pancreatic tumor growth by activating pancreatic stellate cells (PSCs) by interacting with RAGE receptors; however, deficiency in RAGE receptors attenuates the carcinogenic effect. In a study, serum levels of NETs were strongly correlated with the tumor stage of patients with pancreatic duct adenocarcinoma, indicating the potential of NETs as biomarkers in human cancer (65). Dirk et al. discovered that neutrophil infiltration and NET formation were observed in a murine model of nonalcoholic steatohepatitis, followed by the presence of monocyte-derived macrophages, which produced inflammatory cytokines and promoted the progression of hepatocellular carcinoma (HCC) (66).

In addition, NETs can affect the malignant biological behavior of cancers by altering the metabolic characteristics of tumor cells. Yazdani et al. found that NETs controlled mitochondrial homeostasis in tumor cells (67), and inhibition of PAD4 remarkably reduced tumor mitochondrial density and mitochondrial DNA and ATP production and subsequently affected the growth of tumor cells.

NETs can affect the behavioral characteristics of tumor cells by directly activating some receptors and pathways associated with proliferation, migration, and metastasis. In a study, NETs increased cell proliferation and migration of DLBCL cells *in vitro* and tumor growth and lymph node dissemination *in vivo*. Mechanistically, NETs directly upregulated the TLR9 pathway in DLBCL and subsequently triggered the NF-κB, STAT3, and p38 pathways to promote tumor progression. Correspondingly, disruption of NETs or suppression of TLR9 inhibited tumor progression in preclinical models (37). Li et al. found that destruction of the integrity of NETs *in vitro* promoted apoptosis and inhibited invasion and metastasis of GC cells by regulating the Bcl-2, Bax, and NF-κB pathways, suggesting an anti-apoptotic effect of NETs on tumor cells (68).

The invasion and metastasis ability of tumor cells depend on various factors, such as tumor cell motor capacity, angiogenesis, remodeling of the extracellular matrix, and epithelial–mesenchymal transition (EMT). In a study, NETs were found to drive the transformation of typical epithelial morphology to the mesenchymal phenotype and increase the expression of tumor stem cell-related markers in human breast cancer MCF7 cells, which was accompanied by enhanced invasion and migration ability (8). In addition, NET-associated cathepsin G has been reported to facilitate the invasion and metastasis of HCC cells both *in vitro* and *in vivo* (69). The DNA component of NETs can directly act on the CCDC25 receptor on the surface of cancer cells and activates the ILK–β-parvin cascade to enhance the motor capacity of cells (70). Two NET-associated proteases, NE and matrix metalloproteinase 9 (MMP9), can cleave and remodel laminin to reveal an epitope that can subsequently activate FAK/ERK/MLCK/YAP signaling, eventually activating and awakening dormant tumor cells (71).

Furthermore, there is a positive feedback effect between the formation of NETs and CRC progression. For example, NETs can directly promote the secretion of IL-8 from CRC cells, although it can be secreted without NET stimulation (14, 36). As an inflammatory factor, IL-8 can recruit neutrophils and activate neutrophils to produce more NETs, thus aggravating CRC progression. Anquan et al. demonstrated the association between CRC cell-derived exosomes with NETosis, indicating that exosomes originating from KRAS-mutated CRC cells can activate neutrophils to promote NET generation (15), thereby accelerating the deterioration of CRC.

NETs have a close relationship with tumor immune evasion. As major executors of the killing of malignant cells in the tumor immune microenvironment, CD8+ T and natural killer (NK) cells determine the sensitivity of patients to immunotherapy to some extent. NETs may compromise the ability of these cells to kill cancer cells because NET aggregation can enwrap and shield tumor cells to avoid contact with CD8+ T and NK cells (72, 73). Therefore, inhibition of NET formation *via* PAD4 inhibitors may sensitize tumors to immunotherapy.

In addition to tumor progression, the role of NETs in tumor recurrence after treatment is also noteworthy. First, NETs can activate dormant cancer cells by cleaving laminin to remodel ECM in mouse models (71), leading to tumor recurrence. Second, cancer cells can acquire stem cell properties under the influence of NETs (8), and the generation of tumor stem cells is considered an important way through which tumors acquire drug resistance and relapse, which is another way for NETs to promote tumor recurrence.

Despite the carcinogenic effects of NETs revealed in extensive studies, controversial observations have also been reported, such as *in vitro*-produced NETs having anti-tumor effects on CRC. Arelaki et al. found that either PMA or sepsis serum-induced NETs could impede growth and induce apoptosis in CRC cells *in vitro*. However, these inhibitory effects could be abrogated through the destruction of NETs by DNase (74).

### NETs in the Circulatory System

There are several pathways for tumor metastasis including lymphatic, hematogenous metastasis, and implantation metastases, of which hematogenous metastasis is the most common pathway for multiple tumors, such as colorectal liver metastasis. Circulating tumor cells (CTCs) are important for metastasis (75). After detachment from the primary tumor and intravasation, CTCs have to overcome many adverse factors to achieve successful colonization in target organs, a process in which NETs perform a series of functions.

NETs are widely distributed in the blood and physically sequester CTCs as a network structure in the microvessels of distant organs to develop metastasis (76). In addition, Rayes et al. reported that the levels of circulating NETs were higher in patients with multiple cancers, especially those at an advanced stage, than in healthy volunteers (77), indicating the potential role of NETs in distant metastases. However, in addition to physical capture, there may exist certain underlying molecular mechanisms through which NETs can trap CTCs to strengthen their adhesion.

The potential molecular nature of their adhesion was revealed by Najmeh et al. for the first time, and their data shed light on the important role of β1-integrins in mediating the adhesion of tumor cells to NETs (78). In their study, both tumor-derived and NET-derived β1-integrins mediated the adhesion of cancer cells to NETs *in vitro* and *in vivo*. Correspondingly, decreased cancer cell adhesion to liver sinusoids was observed *in vivo* through β1-integrin blockade by its antibody. Considering the multiple effects of NETs on cancer cells, the detailed molecular landscape of interaction between them may be complicated and warrants further investigation.

CTCs are present in the form of single cells and clusters, and the latter possesses greater metastatic capabilities than the former (79). In addition, single cells can form clusters through adhesive molecules in the circulatory system (80). Therefore, we speculated whether NETs affect the formation of clusters from single cells, which can be explained in terms of the following aspects. First, NETs provide scaffolds for the adhesion of CTCs and areas for their encounter, thus providing a spatial basis for the formation of tumor clusters from single cells. In addition, the adhesion of CTCs to NETs partially depends on integrins, which are reported to mediate cell–cell adhesion in tumors (81). CTCs that have successfully attached to NETs should have relatively high expression of integrins on the surface, which constitutes the molecular basis to form clusters. Therefore, we speculate that NETs drive the aggregation of single CTCs into cell clusters, although no direct evidence is available to validate this speculation.

One of the critical steps for the successful colonization of CTCs in target organs is extravasation; however, it is not easy for tumor cells to penetrate the intact blood vessel walls. NETs can facilitate vascular leakage and endothelial-to-mesenchymal transition through elastase-dependent proteolysis of the intercellular junction protein VE-cadherin and activation of β-catenin signaling (82). Therefore, NETs formation within the circulatory system can compromise endothelial integrity, which leaves tumor cells more prone to extravasation. In addition, the presence of NETs decreases the flow rate of CTCs in blood vessels and provides more time for their extravasation and colonization.

### NETs in Liver Metastasis and Beyond

CRC, especially in the advanced stage, is prone to distant metastasis usually in the lung, bone, and liver, among which the most common site of metastasis is the liver (83). This phenomenon can be attributed to many factors. In terms of anatomy, blood from the vein draining the colorectum primarily flows toward the portal vein into the liver, which is also the first site CRC cells reach after detachment. Furthermore, the liver is rich in blood supply and hence provides sufficient nutrition for tumor cell proliferation. Pre-metastatic niche remodeling in the liver is also an important factor, and several studies have indicated the significance of NETs in this process.

Lee et al. reported that NETs could be detected in the omentum of ovarian tumor-bearing mice (TBM) and women with early-stage ovarian cancer without metastasis (84), indicating that NETs formation at the metastatic site occurs before metastasis. The existing NETs at the site capture cancer cells and promote metastasis. To further verify the crucial function of NETs in metastasis, decreased omental metastases were observed in TBM with neutrophil-specific deficiency of PAD4 or those treated with PAD4 pharmacological inhibitors (85). Rayes et al. also observed a significant increase in hepatic adhesion of intrasplenically injected colon cancer cells in TBM compared with non-TBM, DNase1- or NE inhibitor-treated TBM, and PAD4^–/–^ TBM. The results demonstrated that colon primary tumor-induced NETs could promote adhesion of CTC to the liver (77).

Regarding liver metastasis, a study reported that NETs generation in the liver preceded liver metastasis in a breast cancer-bearing mouse model, which occurred earlier than NET formation at the primary tumor site and was elevated in the plasma (70). Studies have also found that the NETs are evident in the liver but have very low levels in other organs, such as the skin and lungs (70, 86), suggesting that NETs favor the liver for metastasis over other sites. This may be partially attributed to the enhanced adhesive capacity of liver sinusoidal endothelial cells (LSECs) to neutrophils, especially during endotoxemia. LSECs can initiate neutrophil adhesion upon the activation of TLR4 receptors by LPS through a CD44/HA/SHAP-dependent mechanism, and Kupffer cells in the liver sinus also contribute to neutrophil recruitment (87). In addition, the liver can retain NETs through the expression of von Willebrand Factor (VWF) in liver sinusoids, which facilitate the adhesion of NETs to LSECs by binding to histones (86).

The formation of liver metastasis can be attributed to the activation of pre-existing dormant cancer cells in the liver, and NETs have been reported to participate in this process. In a murine model of inflammation, induced NETs remodeled ECM *via* NE and MMP9 (71), thus revealing an epitope that triggered dormant cancer cells through integrins and the FAK/ERK/MLCK/YAP signaling pathway. This is also one of the reasons for the postoperative recurrence of cancer.

Neutrophils and NETs can remodel the pre-metastatic niche by facilitating several pathological processes of hepatic diseases (88). Neutrophils are involved in the pathogenesis and progression of alcohol-associated liver disease (ALD); however, they also form an important defense line against infection, which is a leading cause of mortality (89–91). Terence et al. found that NET formation was decreased in a model of ALD, which simultaneously impaired the ability of macrophages to eliminate NETs in the liver, and this impaired ability of clearance aggravates hepatic injury and inflammation (92). Non-alcoholic fatty liver disease (NAFLD) is affected by peripheral metabolic conditions including hepatic fat and insulin resistance (93). Recent studies have implicated that NE participates in the pathogenesis of insulin resistance and obesity (94, 95) and hence influences the progression of NAFLD. Although there is no direct evidence to confirm the impact of NETs on metabolic syndrome and insulin resistance, these studies indicate that some components of NETs can affect the pathogenesis of NAFLD through peripheral mechanisms.

Some clinical studies have shown that neutrophils and NETs are closely related to the liver metastasis of tumors. In patients with HCC or colorectal liver metastasis undergoing hepatectomy (96, 97), a higher neutrophil-to-lymphocyte ratio often predicts a poorer clinical outcome. NETosis induced by postoperative infection promotes hepatic metastasis in a murine model of lung carcinoma (76). Similarly, in a study, a postoperative increase in the level of NETs promoted the progression of liver metastasis in a murine model of liver IR injury and was associated with reduced disease-free survival in a cohort of patients with colorectal liver metastasis undergoing curative liver resection (98).

## Potential Value of NETs for Clinical Application

### Diagnostic Biomarker

At present, liquid biopsy including CTCs, cell-free nucleic acids and extracellular vesicles, has become a promising method for oncology-related examination (99) owing to its characteristics of safety, convenience, and minimal invasion. NETs, which are widely present in the serum, are suitable for detection through liquid biopsy as well. In addition, cfDNA in liquid biopsy, whose clinical diagnostic value has been validated in various tumors (100–102), contains DNA from NETs. However, only a few studies are available to validate the value of solely NETs for tumor diagnosis.

Zhang et al. found that NETs had better diagnostic value than carcinoembryonic antigen (CEA) and carbohydrate antigen 19-9 (CA19-9) in GC by comparing ROC curves (103). In addition, the serum levels of NETs were associated with some clinicopathological features such as lymph node metastasis; however, no relationship was found between the levels of NETs in the tumor tissue and clinicopathological factors. Another study reported that the levels of NETs increased in proportion to the stage of breast cancer, and the levels of NE–DNA complexes were higher in regional and metastatic disease than in localized disease, which was consistent with the speculation that the formation of NETs may result in cancer progression and metastasis (104). However, similar studies on CRC have not yet been reported.

We speculate that, as a new biomarker that can be detected in multiple tumors, NETs may lack specificity used for the diagnosis of a particular tumor type. However, NETs may have better sensitivity and specificity for tumor diagnosis when combined with other tumor-associated markers such as AFP, CEA, and CA199, and their clinical utility should be validated in future studies.

### Therapeutic Target

The role of NETs in the pathogenesis of several tumors and inflammatory diseases has been validated in many studies (6, 105, 106). Therefore, targeting NETs may be of great potential for cancer therapy. NETs can be targeted by blocking the inducers of their formation, inhibiting their formation pathway, destroying their structure, and impeding the tumor–NET interaction (107). Recent studies have reported that therapeutics targeting NETs mainly focus on the following two aspects: inhibition of NETosis and destruction of NET integrity after formation (**Table 1**).

**Table 1:** Published key therapeutics targeting NETs to date.

Category	Treatment	Target and Mechanism	Ref.
Inhibition of NETs formation	Cl-amidine and related compounds	Pan PAD; non-selective irreversible PAD inhibitors	Causey et al. (108)
	GSK-484	PAD4; reversible selective PAD4 inhibitor	Lewis et al. (109)
	BMS-P5	PAD4; selective PAD4 inhibitor	Li et al. (110)
	Kaempferol	ROS‐PAD4 signaling; inhibited ROS production and dsDNA release	Zeng et al. (111)
	Anthracyclines	Suppressed both NADPH oxidase-dependent and -independent NETosis	Khan et al. (112)
	5FU-loaded Amph-PVP nanoparticles	Avoided NETs formation induced by free 5FU	Basyreva et al. (113)
Destruction of NETs’ structural integrity	DNase I	Digested DNA strands	Park et al. (32)
	AAV-mediated gene transfer of DNase I	Increased DNase I secretion and cleave DNA strands	Xia et al. (114), 2020
	DNase-I-coated melanin-like nanosphere	Alleviated NETosis factors; stabilize DNase I in the plasm and prolong its duration of activity	Park et al. (115)
	Thrombomodulin	Degraded HMGB1 derived from NETs and inhibited the induction of NETs	Kajioka et al. (116)
	Recombinant thrombomodulin	Suppressed histone-induced NET release	Shrestha et al. (117)
	5F4 mAb	Blocked CEACAM1 on NETs	Rayes et al. (118)

Extensive efforts have been made to inhibit the generation of NETs pharmacologically to suppress cancer progression. As a critical enzyme in NETosis, PAD4 has been considered the target to block the pro-tumoral effects of NETs. To date, several reports have demonstrated the efficacy of some compounds in inhibiting PAD activity including Cl-amidine, related compounds that are irreversible non-selective PAD inhibitors (108, 119), and the reversible selective PAD4 inhibitor GSK-484 (109). Another small-molecule inhibitor, BMS-P5, was demonstrated to abrogate NET formation induced by multiple myeloma cells and delay disease progression in a murine model (110). In addition, some clinical antitumor drugs affect NET formation. Zeng et al. found that kaempferol, whose inhibitory effects on primary tumor growth have been widely investigated, can suppress NETosis by inhibiting the ROS–PAD4 pathway (111). Anthracyclines, ranked among the most effective chemotherapeutics against cancer that act through DNA intercalation, oxidative stress, and topoisomerase II poisoning (120), suppress both NADPH oxidase-dependent and independent NETosis in human neutrophils (112), which further elucidates the antitumor mechanism of anthracyclines. Similarly, free 5FU, a chemotherapeutic agent, leads to a significant and rapid increase in the total amount of NETs in the blood. However, when 5FU-loaded amphiphilic poly-N-vinylpyrrolidone nanoparticles were studied under the same conditions, the level of NETs in the blood was not elevated, indicating the significant potential of nanoparticles as delivery agents for chemotherapeutic drugs in antitumor therapy (113).

Another way to suppress the pro-tumoral effects of NETs is to destroy their structural integrity by targeting DNA, comprising their backbone and affiliated proteins. DNase I has antimetastatic activity and can inhibit NETs (32); however, its short biological half-life limits its clinical utility. Xia et al. demonstrated that adeno-associated virus-mediated DNase I liver gene transfer inhibited neutrophil infiltration and NET formation, increases the proportion of CD8^+^ T cells at the tumor site, and eventually attenuates the progression of liver metastasis in a mouse model of colorectal liver metastasis (114), representing a novel and effective therapeutic strategy for CRC. A recent study reported that DNase-I-coated melanin-like nanospheres promoted dissolution of NET structure in sepsis-associated NETosis, thereby preventing further progression of the disease; however, its antitumor effects warrant further investigation (115). Some protein components of NETs play a significant role in the development of tumors, which can also be targeted to impair NET function. In a study, HMGB1 derived from NETs strengthened the malignancy of cancer cells in a mouse model of liver metastasis with inflammation. However, thrombomodulin (TM) potentiated HMGB1 proteolysis *via* thrombin and consequently inhibited the induction of NETs, thereby preventing pancreatic cancer metastasis to the liver (116). In addition, recombinant TM (rTM) can suppress histone-induced NET release both *in vitro* and *in vivo* by binding to histones (117). Rayes et al. discovered that NET-associated carcinoembryonic Ag cell adhesion molecule 1 (CEACAM1) was an important element for tumor progression and metastasis, and blocking CEACAM1 on NETs or its knockout in a mouse model significantly compromised cell adhesion, migration and metastasis in colon carcinoma (118). In a recent study, a novel method was introduced to use NETs as anti-tumor drug delivery vehicles by re-engineering neutrophils to express the apoptosis-inducing chimeric eGFP-TRAIL protein on NETs, which can simultaneously entrap and kill tumor cells while reserving their antibacterial capabilities (121), making NETs a promising candidate for the delivery of antitumoral agents.

Furthermore, NETs are associated with therapy resistance. Inhibition of neutrophils or PAD4-dependent NETosis can increase sensitivity to immune checkpoint blockade in pancreatic cancer (122), indicating that NETs are a potential candidate for improving immunotherapeutic efficacy.

### Prognostic Prediction

The pro-tumoral effects of NETs are suggestive of their potential as a novel prognostic predictor of cancers (123), which has been reported in several studies. Elevated levels of NETs are strongly associated with a higher risk of metastasis. Decker et al. showed that increased NETosis in blood could be used as a biomarker to detect early head and neck cancer and predict the possibility of developing tumor metastasis (124).

Furthermore, increased levels of NETs have a strong correlation with unfavorable survival in many types of tumors. For example, higher preoperative serum NET levels were closely associated with shorter recurrence-free survival (RFS) and overall survival (OS) in patients with primary hepatic malignancies (125). In addition, preoperative moderate leucocytosis is correlated with increased levels of tumor-infiltrating NETs in esophageal cancer (EC), which is associated with worse OS and disease-free survival (DFS). The level of NETs is considered an independent prognostic factor for survival in EC after surgery (126). In patients with GC, higher baseline levels of NETs in the blood are significantly correlated with worse progression-free survival (PFS) (103). However, in a study on patients with high-grade ovarian cancer, a contradictory conclusion was proposed, indicating that NETs are related to favorable survival and better outcomes (127).

In addition, the level of NETs in the blood can be used to predict the effectiveness of treatment regimens in patients with cancer. Zhang et al. found that the level of NETs was inversely correlated with short-term therapeutic efficacy in patients with GC who had received advanced first-line treatment (103). This study indicated the possibility of enhanced chemotherapeutic efficacy through NETosis inhibition.

Venous thromboembolism (VTE), a common complication in patients with cancer, can be induced and aggravated by NET formation (128). Extensive studies have confirmed the relationship between increased NET levels and a higher risk of thrombosis in many diseases including cancer (50, 129), which indicates the involvement of NETs in cancer-associated thrombosis and the significance of NETs as prognostic biomarkers to predict the risk of thromboembolism.

## Conclusion and Perspectives

Several studies have validated the role of NETs in tumorigenesis, metastasis spread, and associated complications, indicating the significant potential of targeting NETs for cancer therapy. On the one hand, further investigation is required to study the detailed molecular mechanisms of NETs formation and pro-tumoral pathways affected by NETs to identify more therapeutic targets and develop corresponding agents. On the other hand, solely inhibiting overall NET formation may compromise immunity because NETosis is a part of the immune system. Therefore, it is necessary to develop therapeutics precisely targeting NETs in tumor tissues but without adverse effects on immune function. At present, a gene therapy vector has been reported to specifically express DNase in the liver and effectively inhibit colorectal liver metastasis, which indicates the possibility of achieving tumor precision therapy targeting NETs.

NETs can be used for tumor diagnosis and prognosis combined with some classical tumor markers; however, the results may not necessarily be reliable, limited by sample number, species specificity, and other unknown factors. Therefore, large-scale, multicenter studies should be performed to further verify the potential of NETs as diagnostic and prognostic biomarkers. In addition, fecal testing is currently one of the key methods for early CRC screening. Therefore, could NETs be present in the stool of patients with CRC and considered an early screening indicator?

NETs are involved in inflammation in chronic liver diseases. In patients with CRC, increased levels of NETs in the liver often indicate a high metastatic rate. Therefore, the management of chronic liver disease in patients with CRC is very important. Controlling the levels of NETs in the liver as early as possible may prevent or decrease metastasis. In addition, developing a method for rapid, minimally invasive, inexpensive, and stable detection of NETs is the basis for further clinical utility; however, the currently available detection technology cannot meet these requirements, which should be further improved.

CRC refers to colon cancer, including left and right colon cancer, and rectal cancer, which have different molecular landscapes in tumorigenesis despite a close relationship. Therefore, the role of NETs in tumorigenesis is also different and should be investigated in future studies.

## Author Contributions

SJ and GD: conception of the work. DL, JS and BC: literature search and manuscript drafting. PC, LJ and LC: design of the table and figures. RZ, HL, and WG: critical revision of the work and final version approval. All authors contributed to the article and approved the submitted version.

## Conflict of Interest

The authors declare that the research was conducted in the absence of any commercial or financial relationships that could be construed as a potential conflict of interest.

## Publisher’s Note

All claims expressed in this article are solely those of the authors and do not necessarily represent those of their affiliated organizations, or those of the publisher, the editors and the reviewers. Any product that may be evaluated in this article, or claim that may be made by its manufacturer, is not guaranteed or endorsed by the publisher.

## References

[1] Lehman HK Segal BH. The Role of Neutrophils in Host Defense and Disease. J Allergy Clin Immunol (2020) 145(6):1535–44. doi: 10.1016/j.jaci.2020.02.038 PMC8912989 32283205

[2] Dąbrowska D Jabłońska E Iwaniuk A Garley M. Many Ways-One Destination: Different Types of Neutrophils Death. Int Rev Immunol (2019) 38(1):18–32. doi: 10.1080/08830185.2018.1540616 30516403

[3] Brinkmann V Reichard U Goosmann C Fauler B Uhlemann Y Weiss DS. Neutrophil Extracellular Traps Kill Bacteria. Science (2004) 303(5663):1532–5. doi: 10.1126/science.1092385 15001782

[4] Delgado-Rizo V Martínez-Guzmán MA Iñiguez-Gutierrez L García-Orozco A Alvarado-Navarro A Fafutis-Morris M. Neutrophil Extracellular Traps and Its Implications in Inflammation: An Overview. Front Immunol (2017) 8:81. doi: 10.3389/fimmu.2017.00081 28220120 PMC5292617

[5] Lee KH Kronbichler A Park DD Park Y Moon H Kim H. Neutrophil Extracellular Traps (Nets) in Autoimmune Diseases: A Comprehensive Review. Autoimmun Rev (2017) 16(11):1160–73. doi: 10.1016/j.autrev.2017.09.012 28899799

[6] Barnes BJ Adrover JM Baxter-Stoltzfus A Borczuk A Cools-Lartigue J Crawford JM. Targeting Potential Drivers of Covid-19: Neutrophil Extracellular Traps. J Exp Med (2020) 217(6):e20200652. doi: 10.1084/jem.20200652 32302401 PMC7161085

[7] Zha C Meng X Li L Mi S Qian D Li Z. Neutrophil Extracellular Traps Mediate the Crosstalk Between Glioma Progression and the Tumor Microenvironment *Via* the Hmgb1/Rage/Il-8 Axis. Cancer Biol Med (2020) 17(1):154–68. doi: 10.20892/j.issn.2095-3941.2019.0353 PMC7142852 32296583

[8] Martins-Cardoso K Almeida VH Bagri KM Rossi MID Mermelstein CS Konig S. Neutrophil Extracellular Traps (Nets) Promote Pro-Metastatic Phenotype in Human Breast Cancer Cells Through Epithelial-Mesenchymal Transition. Cancers (Basel) (2020) 12(6):1542. doi: 10.3390/cancers12061542 PMC7352979 32545405

[9] Schedel F Mayer-Hain S Pappelbaum KI Metze D Stock M Goerge T. Evidence and Impact of Neutrophil Extracellular Traps in Malignant Mel Anoma. Pigment Cell Melanoma Res (2020) 33(1):63–73. doi: 10.1111/pcmr.12818 31402559

[10] Siegel RL Miller KD Jemal A. Cancer Statistics, 2019. CA Cancer J Clin (2019) 69(1):7–34. doi: 10.3322/caac.21551 30620402

[11] Jin M Frankel WL. Lymph Node Metastasis in Colorectal Cancer. Surg Oncol Clin N Am (2018) 27(2):401–12. doi: 10.1016/j.soc.2017.11.011 29496097

[12] Tsikitis VL Larson DW Huebner M Lohse CM Thompson PA. Predictors of Recurrence Free Survival for Patients With Stage Ii and Iii Colon Cancer. BMC Cancer (2014) 14:336. doi: 10.1186/1471-2407-14-336 24886281 PMC4029910

[13] Miyamoto Y Hayashi N Sakamoto Y Ohuchi M Tokunagam R Kurashige J. Predictors of Long-Term Survival in Patients With Stage Iv Colorectal Cancer With Multi-Organ Metastases: A Single-Center Retrospective Analysis. Int J Clin Oncol (2015) 20(6):1140–6. doi: 10.1007/s10147-015-0835-2 25947545

[14] Yang L Liu L Zhang R Hong J Wang Y Wang J. Il-8 Mediates a Positive Loop Connecting Increased Neutrophil Extracellular Traps (Nets) and Colorectal Cancer Liver Metastasis. J Cancer (2020) 11(15):4384–96. doi: 10.7150/jca.44215 PMC7255375 32489457

[15] Shang A Gu C Zhou C Yang Y Chen C Zeng B. Exosomal Kras Mutation Promotes the Formation of Tumor-Associated Neutrophil Extracellular Traps and Causes Deterioration of Colorectal Cancer by Inducing Il-8 Expression. Cell Commun Signal (2020) 18(1):52. doi: 10.1186/s12964-020-0517-1 32228650 PMC7106821

[16] Khan U Chowdhury S Billah MM Islam KMD Thorlacius H Rahman M. Neutrophil Extracellular Traps in Colorectal Cancer Progression and Metastasis. Int J Mol Sci (2021) 22(14):7260. doi: 10.3390/ijms22147260 34298878 PMC8307027

[17] Jorch SK Kubes P. An Emerging Role for Neutrophil Extracellular Traps in Noninfectious Disease. Nat Med (2017) 23(3):279–87. doi: 10.1038/nm.4294 28267716

[18] Rochael NC Guimaraes-Costa AB Nascimento MT DeSouza-Vieira TS Oliveira MP Garcia e Souza LF. Classical Ros-Dependent and Early/Rapid Ros-Independent Release of Neutrophil Extracellular Traps Triggered by Leishmania Parasites. Sci Rep (2015) 5:18302. doi: 10.1038/srep18302 26673780 PMC4682131

[19] Lin AM Rubin CJ Khandpur R Wang JY Riblett M Yalavarthi S. Mast Cells and Neutrophils Release Il-17 Through Extracellular Trap Formation in Psoriasis. J Immunol (2011) 187(1):490–500. doi: 10.4049/jimmunol.1100123 21606249 PMC3119764

[20] Grayson PC Kaplan MJ. At the Bench: Neutrophil Extracellular Traps (Nets) Highlight Novel Aspects of Innate Immune System Involvement in Autoimmune Diseases. J Leukoc Biol (2016) 99(2):253–64. doi: 10.1189/jlb.5BT0615-247R PMC4718195 26432901

[21] Clark SR Ma AC Tavener SA McDonald B Goodarzi Z Kelly MM. Platelet Tlr4 Activates Neutrophil Extracellular Traps to Ensnare Bacteria in Septic Blood. Nat Med (2007) 13(4):463–9. doi: 10.1038/nm1565 17384648

[22] Kirchner T Moller S Klinger M Solbach W Laskay T Behnen M. The Impact of Various Reactive Oxygen Species on the Formation of Neutrophil Extracellular Traps. Mediators Inflamm (2012) 2012:849136. doi: 10.1155/2012/849136 22481865 PMC3317033

[23] Warnatsch A Tsourouktsoglou TD Branzk N Wang Q Reincke S Herbst S. Reactive Oxygen Species Localization Programs Inflammation to Clear Microbes of Different Size. Immunity (2017) 46(3):421–32. doi: 10.1016/j.immuni.2017.02.013 PMC5965455 28314592

[24] Joshi MB Philippova M Ivanov D Allenspach R Erne P Resink TJ. T-Cadherin Protects Endothelial Cells From Oxidative Stress-Induced Apoptosis. FASEB J (2005) 19(12):1737–9. doi: 10.1096/fj.05-3834fje 16099944

[25] Gupta AK Joshi MB Philippova M Erne P Hasler P Hahn S. Activated Endothelial Cells Induce Neutrophil Extracellular Traps and Are Susceptible to Netosis-Mediated Cell Death. FEBS Lett (2010) 584(14):3193–7. doi: 10.1016/j.febslet.2010.06.006 20541553

[26] Jeannin P Delneste Y Gosset P Molet S Lassalle P Hamid Q. Histamine Induces Interleukin-8 Secretion by Endothelial Cells. Blood (1994) 84(7):2229–33. doi: 10.1182/blood.V84.7.2229.2229 7919340

[27] Maugeri N Campana L Gavina M Covino C De Metrio M Panciroli C. Activated Platelets Present High Mobility Group Box 1 to Neutrophils, Inducing Autophagy and Promoting the Extrusion of Neutrophil Extracellular Traps. J Thromb Haemost (2014) 12(12):2074–88. doi: 10.1111/jth.12710 25163512

[28] Etulain J Martinod K Wong SL Cifuni SM Schattner M Wagner DD. P-Selectin Promotes Neutrophil Extracellular Trap Formation in Mice. Blood (2015) 126(2):242–6. doi: 10.1182/blood-2015-01-624023 PMC4497964 25979951

[29] Li J Kim K Hahm E Molokie R Hay N Gordeuk VR. Neutrophil Akt2 Regulates Heterotypic Cell-Cell Interactions During Vascular Inflammation. J Clin Invest (2014) 124(4):1483–96. doi: 10.1172/JCI72305 PMC3973084 24642468

[30] Sreeramkumar V Adrover JM Ballesteros I Cuartero MI Rossaint J Bilbao I. Neutrophils Scan for Activated Platelets to Initiate Inflammation. Science (2014) 346(6214):1234–8. doi: 10.1126/science.1256478 PMC4280847 25477463

[31] Andrews RK Arthur JF Gardiner EE. Neutrophil Extracellular Traps (Nets) and the Role of Platelets in Infection. Thromb Haemost (2014) 112(4):659–65. doi: 10.1160/TH14-05-0455 25265341

[32] Park J Wysocki RW Amoozgar Z Maiorino L Fein MR Jorns J. Cancer Cells Induce Metastasis-Supporting Neutrophil Extracellular DNA Traps. Sci Transl Med (2016) 8(361):361ra138. doi: 10.1126/scitranslmed.aag1711 PMC5550900 27798263

[33] DuPre SA Hunter KW Jr. Murine Mammary Carcinoma 4t1 Induces a Leukemoid Reaction With Splenomegaly: Association With Tumor-Derived Growth Factors. Exp Mol Pathol (2007) 82(1):12–24. doi: 10.1016/j.yexmp.2006.06.007 16919266

[34] Kowanetz M Wu X Lee J Tan M Hagenbeek T Qu X. Granulocyte-Colony Stimulating Factor Promotes Lung Metastasis Through Mobilization of Ly6g+Ly6c+ Granulocytes. Proc Natl Acad Sci U.S.A. (2010) 107(50):21248–55. doi: 10.1073/pnas.1015855107 PMC3003076 21081700

[35] Demers M Krause DS Schatzberg D Martinod K Voorhees JR Fuchs TA. Cancers Predispose Neutrophils to Release Extracellular DNA Traps That Contribute to Cancer-Associated Thrombosis. Proc Natl Acad Sci USA (2012) 109(32):13076–81. doi: 10.1073/pnas.1200419109 PMC3420209 22826226

[36] Alfaro C Teijeira A Oñate C Pérez G Sanmamed MF Andueza MP. Tumor-Produced Interleukin-8 Attracts Human Myeloid-Derived Suppressor Cells and Elicits Extrusion of Neutrophil Extracellular Traps (Nets). Clin Cancer Res (2016) 22(15):3924–36. doi: 10.1158/1078-0432.ccr-15-2463 26957562

[37] Nie M Yang L Bi X Wang Y Sun P Yang H. Neutrophil Extracellular Traps Induced by Il8 Promote Diffuse Large B-Cell Lymphoma Progression *Via* the Tlr9 Signaling. Clin Cancer Res (2019) 25(6):1867–79. doi: 10.1158/1078-0432.ccr-18-1226 30446590

[38] Liu K Sun E Lei M Li L Gao J Nian X. Bcg-Induced Formation of Neutrophil Extracellular Traps Play an Important Role in Bladder Cancer Treatment. Clin Immunol (2019) 201:4–14. doi: 10.1016/j.clim.2019.02.005 30771501

[39] Gomes T Várady CBS Lourenço AL Mizurini DM Rondon AMR Leal AC. Il-1β Blockade Attenuates Thrombosis in a Neutrophil Extracellular Trap-Dependent Breast Cancer Model. Front Immunol (2019) 10:2088. doi: 10.3389/fimmu.2019.02088 31552036 PMC6737452

[40] Jin W Yin H Li H Yu XJ Xu HX Liu L. Neutrophil Extracellular DNA Traps Promote Pancreatic Cancer Cells Migration and Invasion by Activating Egfr/Erk Pathway. J Cell Mol Med (2021) 25(12):5443–56. doi: 10.1111/jcmm.16555 PMC8184670 33955688

[41] Chang X Han J Pang L Zhao Y Yang Y Shen Z. Increased Padi4 Expression in Blood and Tissues of Patients With Malignant Tumors. BMC Cancer (2009) 9:40. doi: 10.1186/1471-2407-9-40 19183436 PMC2637889

[42] Akers JC Gonda D Kim R Carter BS Chen CC. Biogenesis of Extracellular Vesicles (Ev): Exosomes, Microvesicles, Retrovirus-Like Vesicles, and Apoptotic Bodies. J Neurooncol (2013) 113(1):1–11. doi: 10.1007/s11060-013-1084-8 23456661 PMC5533094

[43] Leal AC Mizurini DM Gomes T Rochael NC Saraiva EM Dias MS. Tumor-Derived Exosomes Induce the Formation of Neutrophil Extracellular Traps: Implications for the Establishment of Cancer-Associated Thrombosis. Sci Rep (2017) 7(1):6438. doi: 10.1038/s41598-017-06893-7 28743887 PMC5526939

[44] Becker A Thakur BK Weiss JM Kim HS Peinado H Lyden D. Extracellular Vesicles in Cancer: Cell-To-Cell Mediators of Metastasis. Cancer Cell (2016) 30(6):836–48. doi: 10.1016/j.ccell.2016.10.009 PMC5157696 27960084

[45] Chennakrishnaiah S Meehan B D'Asti E Montermini L Lee TH Karatzas N. Leukocytes as a Reservoir of Circulating Oncogenic DNA and Regulatory Targets of Tumor-Derived Extracellular Vesicles. J Thromb Haemost (2018) 16(9):1800–13. doi: 10.1111/jth.14222 29971917

[46] Aguila S de Los Reyes-Garcia AM Fernandez-Perez MP Reguilon-Gallego L Zapata-Martinez L Ruiz-Lorente I. Micrornas as New Regulators of Neutrophil Extracellular Trap Formation. Int J Mol Sci (2021) 22(4):2116. doi: 10.3390/ijms22042116 33672737 PMC7924615

[47] Arroyo AB de Los Reyes-García AM Rivera-Caravaca JM Valledor P García-Barberá N Roldán V. Mir-146a Regulates Neutrophil Extracellular Trap Formation That Predicts Adverse Cardiovascular Events in Patients With Atrial Fibrillation. Arterioscler Thromb Vasc Biol (2018) 38(4):892–902. doi: 10.1161/atvbaha.117.310597 29437577

[48] Hawez A Al-Haidari A Madhi R Rahman M Thorlacius H. Mir-155 Regulates Pad4-Dependent Formation of Neutrophil Extracellular Traps. Front Immunol (2019) 10:2462. doi: 10.3389/fimmu.2019.02462 31736940 PMC6838784

[49] Brill A Fuchs TA Chauhan AK Yang JJ De Meyer SF Kollnberger M. Von Willebrand Factor-Mediated Platelet Adhesion Is Critical for Deep Vein Thrombosis in Mouse Models. Blood (2011) 117(4):1400–7. doi: 10.1182/blood-2010-05-287623 PMC3056477 20959603

[50] Fuchs TA Brill A Duerschmied D Schatzberg D Monestier M Myers DD Jr. Extracellular DNA Traps Promote Thrombosis. Proc Natl Acad Sci USA (2010) 107(36):15880–5. doi: 10.1073/pnas.1005743107 PMC2936604 20798043

[51] Kessenbrock K Krumbholz M Schönermarck U Back W Gross WL Werb Z. Netting Neutrophils in Autoimmune Small-Vessel Vasculitis. Nat Med (2009) 15(6):623–5. doi: 10.1038/nm.1959 PMC2760083 19448636

[52] Khandpur R Carmona-Rivera C Vivekanandan-Giri A Gizinski A Yalavarthi S Knight JS. Nets Are a Source of Citrullinated Autoantigens and Stimulate Inflammatory Responses in Rheumatoid Arthritis. Sci Transl Med (2013) 5(178):178ra40. doi: 10.1126/scitranslmed.3005580 PMC3727661 23536012

[53] Garcia-Romo GS Caielli S Vega B Connolly J Allantaz F Xu Z. Netting Neutrophils Are Major Inducers of Type I Ifn Production in Pediatric Systemic Lupus Erythematosus. Sci Transl Med (2011) 3(73):73ra20. doi: 10.1126/scitranslmed.3001201 PMC3143837 21389264

[54] Warnatsch A Ioannou M Wang Q Papayannopoulos V. Inflammation. Neutrophil Extracellular Traps License Macrophages for Cytokine Production in Atherosclerosis. Science (2015) 349(6245):316–20. doi: 10.1126/science.aaa8064 PMC4854322 26185250

[55] Huang H Tohme S Al-Khafaji AB Tai S Loughran P Chen L. Damage-Associated Molecular Pattern-Activated Neutrophil Extracellular Trap Exacerbates Sterile Inflammatory Liver Injury. Hepatology (2015) 62(2):600–14. doi: 10.1002/hep.27841 PMC4515210 25855125

[56] Zenaro E Pietronigro E Della Bianca V Piacentino G Marongiu L Budui S. Neutrophils Promote Alzheimer's Disease-Like Pathology and Cognitive Decline *Via* Lfa-1 Integrin. Nat Med (2015) 21(8):880–6. doi: 10.1038/nm.3913 26214837

[57] Dinallo V Marafini I Di Fusco D Laudisi F Franzè E Di Grazia A. Neutrophil Extracellular Traps Sustain Inflammatory Signals in Ulcerative Colitis. J Crohns Colitis (2019) 13(6):772–84. doi: 10.1093/ecco-jcc/jjy215 30715224

[58] Yashiro M. Ulcerative Colitis-Associated Colorectal Cancer. World J Gastroenterol (2014) 20(44):16389–97. doi: 10.3748/wjg.v20.i44.16389 PMC4248182 25469007

[59] D'Abbondanza M Martorelli EE Ricci MA De Vuono S Migliola EN Godino C. Increased Plasmatic Nets by-Products in Patients in Severe Obesity. Sci Rep (2019) 9(1):14678. doi: 10.1038/s41598-019-51220-x 31604985 PMC6789039

[60] Freitas DF Colón DF Silva RL Santos EM Guimarães VHD Ribeiro GHM. Neutrophil Extracellular Traps (Nets) Modulate Inflammatory Profile in Obese Humans and Mice: Adipose Tissue Role on Nets Levels. Mol Biol Rep (2022) 49(4):3225–36. doi: 10.1007/s11033-022-07157-y 35066770

[61] Wong SL Demers M Martinod K Gallant M Wang Y Goldfine AB. Diabetes Primes Neutrophils to Undergo Netosis, Which Impairs Wound Healing. Nat Med (2015) 21(7):815–9. doi: 10.1038/nm.3887 PMC4631120 26076037

[62] Sung H Siegel RL Rosenberg PS Jemal A. Emerging Cancer Trends Among Young Adults in the USA: Analysis of a Population-Based Cancer Registry. Lancet Public Health (2019) 4(3):e137–47. doi: 10.1016/s2468-2667(18)30267-6 30733056

[63] De Bruijn KM Arends LR Hansen BE Leeflang S Ruiter R van Eijck CH. Systematic Review and Meta-Analysis of the Association Between Diabetes Mellitus and Incidence and Mortality in Breast and Colorectal Cancer. Br J Surg (2013) 100(11):1421–9. doi: 10.1002/bjs.9229 24037561

[64] Richardson JJR Hendrickse C Gao-Smith F Thickett DR. Neutrophil Extracellular Trap Production in Patients With Colorectal Cancer *in Vitro* . Int J Inflam (2017) 2017:4915062. doi: 10.1155/2017/4915062 28828191 PMC5554570

[65] Miller-Ocuin JL Liang X Boone BA Doerfler WR Singhi AD Tang D. DNA Released From Neutrophil Extracellular Traps (Nets) Activates Pancreatic Stellate Cells and Enhances Pancreatic Tumor Growth. Oncoimmunology (2019) 8(9):e1605822. doi: 10.1080/2162402X.2019.1605822 31428515 PMC6685506

[66] van der Windt DJ Sud V Zhang H Varley PR Goswami J Yazdani HO. Neutrophil Extracellular Traps Promote Inflammation and Development of Hepatocellular Carcinoma in Nonalcoholic Steatohepatitis. Hepatology (2018) 68(4):1347–60. doi: 10.1002/hep.29914 PMC6173613 29631332

[67] Yazdani HO Roy E Comerci AJ van der Windt DJ Zhang H Huang H. Neutrophil Extracellular Traps Drive Mitochondrial Homeostasis in Tumors to Augment Growth. Cancer Res (2019) 79(21):5626–39. doi: 10.1158/0008-5472.CAN-19-0800 PMC6825588 31519688

[68] Li R Zou X Zhu T Xu H Li X Zhu L. Destruction of Neutrophil Extracellular Traps Promotes the Apoptosis and Inhibits the Invasion of Gastric Cancer Cells by Regulating the Expression of Bcl-2, Bax and Nf-Kappab. Onco Targets Ther (2020) 13:5271–81. doi: 10.2147/OTT.S227331 PMC7293391 32606746

[69] Guan X Lu Y Zhu H Yu S Zhao W Chi X. The Crosstalk Between Cancer Cells and Neutrophils Enhances Hepatocellular Carcinoma Metastasis *Via* Neutrophil Extracellular Traps-Associated Cathepsin G Component: A Potential Therapeutic Target. J Hepatocell Carcinoma (2021) 8:451–65. doi: 10.2147/jhc.s303588 PMC8144903 34046369

[70] Yang L Liu Q Zhang X Liu X Zhou B Chen J. DNA of Neutrophil Extracellular Traps Promotes Cancer Metastasis *Via* Ccdc25. Nature (2020) 583(7814):133–8. doi: 10.1038/s41586-020-2394-6 32528174

[71] Albrengues J Shields MA Ng D Park CG Ambrico A Poindexter ME. Neutrophil Extracellular Traps Produced During Inflammation Awaken Dormant Cancer Cells in Mice. Science (2018) 361(6409):eaao4227. doi: 10.1126/science.aao4227 30262472 PMC6777850

[72] Teijeira Á Garasa S Gato M Alfaro C Migueliz I Cirella A. Cxcr1 and Cxcr2 Chemokine Receptor Agonists Produced by Tumors Induce Neutrophil Extracellular Traps That Interfere With Immune Cytotoxicity. Immunity (2020) 52(5):856–71.e8. doi: 10.1016/j.immuni.2020.03.001 32289253

[73] Ireland AS Oliver TG. Neutrophils Create an Impenetrable Shield Between Tumor and Cytotoxic Immune Cells. Immunity (2020) 52(5):729–31. doi: 10.1016/j.immuni.2020.04.009 PMC7851833 32433945

[74] Arelaki S Arampatzioglou A Kambas K Papagoras C Miltiades P Angelidou I. Gradient Infiltration of Neutrophil Extracellular Traps in Colon Cancer and Evidence for Their Involvement in Tumour Growth. PloS One (2016) 11(5):e0154484. doi: 10.1371/journal.pone.0154484 27136460 PMC4852909

[75] Rodrigues P Vanharanta S. Circulating Tumor Cells: Come Together, Right Now, Over Metastasis. Cancer Discov (2019) 9(1):22–4. doi: 10.1158/2159-8290.cd-18-1285 PMC6420083 30626605

[76] Cools-Lartigue J Spicer J McDonald B Gowing S Chow S Giannias B. Neutrophil Extracellular Traps Sequester Circulating Tumor Cells and Promote Metastasis. J Clin Invest (2013) 123(8):3446–58. doi: 10.1172/JCI67484 PMC3726160 23863628

[77] Rayes RF Mouhanna JG Nicolau I Bourdeau F Giannias B Rousseau S. Primary Tumors Induce Neutrophil Extracellular Traps With Targetable Metastasis Promoting Effects. JCI Insight (2019) 5(16):e128008. doi: 10.1172/jci.insight.128008 PMC6777835 31343990

[78] Najmeh S Cools-Lartigue J Rayes RF Gowing S Vourtzoumis P Bourdeau F. Neutrophil Extracellular Traps Sequester Circulating Tumor Cells *Via* B1-Integrin Mediated Interactions. Int J Cancer (2017) 140(10):2321–30. doi: 10.1002/ijc.30635 28177522

[79] Amintas S Bedel A Moreau-Gaudry F Boutin J Buscail L Merlio JP. Circulating Tumor Cell Clusters: United We Stand Divided We Fall. Int J Mol Sci (2020) 21(7):2653. doi: 10.3390/ijms21072653 PMC7177734 32290245

[80] Liu X Taftaf R Kawaguchi M Chang YF Chen W Entenberg D. Homophilic Cd44 Interactions Mediate Tumor Cell Aggregation and Polyclonal Metastasis in Patient-Derived Breast Cancer Models. Cancer Discovery (2019) 9(1):96–113. doi: 10.1158/2159-8290.cd-18-0065 30361447 PMC6328322

[81] An T Zhang Z Li Y Yi J Zhang W Chen D. Integrin B1-Mediated Cell^-^Cell Adhesion Augments Metformin-Induced Anoikis. Int J Mol Sci (2019) 20(5):1161. doi: 10.3390/ijms20051161 PMC6429125 30866414

[82] Pieterse E Rother N Garsen M Hofstra JM Satchell SC Hoffmann M. Neutrophil Extracellular Traps Drive Endothelial-To-Mesenchymal Transition. Arterioscler Thromb Vasc Biol (2017) 37(7):1371–9. doi: 10.1161/atvbaha.117.309002 28495931

[83] Teng S Li YE Yang M Qi R Huang Y Wang Q. Tissue-Specific Transcription Reprogramming Promotes Liver Metastasis of Colorectal Cancer. Cell Res (2020) 30(1):34–49. doi: 10.1038/s41422-019-0259-z 31811277 PMC6951341

[84] Lee W Ko SY Mohamed MS Kenny HA Lengyel E Naora H. Neutrophils Facilitate Ovarian Cancer Premetastatic Niche Formation in the Omentum. J Exp Med (2019) 216(1):176–94. doi: 10.1084/jem.20181170 PMC6314534 30567719

[85] Lee W Naora H. Neutrophils Fertilize the Pre-Metastatic Niche. Aging (Albany NY) (2019) 11(17):6624–5. doi: 10.18632/aging.102258 PMC6756893 31509520

[86] Kolaczkowska E Jenne CN Surewaard BG Thanabalasuriar A Lee WY Sanz MJ. Molecular Mechanisms of Net Formation and Degradation Revealed by Intravital Imaging in the Liver Vasculature. Nat Commun (2015) 6:6673. doi: 10.1038/ncomms7673 25809117 PMC4389265

[87] McDonald B Jenne CN Zhuo L Kimata K Kubes P. Kupffer Cells and Activation of Endothelial Tlr4 Coordinate Neutrophil Adhesion Within Liver Sinusoids During Endotoxemia. Am J Physiol Gastrointest Liver Physiol (2013) 305(11):G797–806. doi: 10.1152/ajpgi.00058.2013 24113769

[88] Hilscher MB Shah VH. Neutrophil Extracellular Traps and Liver Disease. Semin Liver Dis (2020) 40(2):171–9. doi: 10.1055/s-0039-3399562 PMC7195236 31726473

[89] Louvet A Wartel F Castel H Dharancy S Hollebecque A Canva-Delcambre V. Infection in Patients With Severe Alcoholic Hepatitis Treated With Steroids: Early Response to Therapy Is the Key Factor. Gastroenterology (2009) 137(2):541–8. doi: 10.1053/j.gastro.2009.04.062 19445945

[90] Michelena J Altamirano J Abraldes JG Affò S Morales-Ibanez O Sancho-Bru P. Systemic Inflammatory Response and Serum Lipopolysaccharide Levels Predict Multiple Organ Failure and Death in Alcoholic Hepatitis. Hepatology (2015) 62(3):762–72. doi: 10.1002/hep.27779 PMC4549175 25761863

[91] Taylor NJ Nishtala A Manakkat Vijay GK Abeles RD Auzinger G Bernal W. Circulating Neutrophil Dysfunction in Acute Liver Failure. Hepatology (2013) 57(3):1142–52. doi: 10.1002/hep.26102 23079896

[92] Bukong TN Cho Y Iracheta-Vellve A Saha B Lowe P Adejumo A. Abnormal Neutrophil Traps and Impaired Efferocytosis Contribute to Liver Injury and Sepsis Severity After Binge Alcohol Use. J Hepatol (2018) 69(5):1145–54. doi: 10.1016/j.jhep.2018.07.005 PMC6310218 30030149

[93] van der Poorten D Milner KL Hui J Hodge A Trenell MI Kench JG. Visceral Fat: A Key Mediator of Steatohepatitis in Metabolic Liver Disease. Hepatology (2008) 48(2):449–57. doi: 10.1002/hep.22350 18627003

[94] Talukdar S Oh DY Bandyopadhyay G Li D Xu J McNelis J. Neutrophils Mediate Insulin Resistance in Mice Fed a High-Fat Diet Through Secreted Elastase. Nat Med (2012) 18(9):1407–12. doi: 10.1038/nm.2885 PMC3491143 22863787

[95] Mansuy-Aubert V Zhou QL Xie X Gong Z Huang JY Khan AR. Imbalance Between Neutrophil Elastase and Its Inhibitor A1-Antitrypsin in Obesity Alters Insulin Sensitivity, Inflammation, and Energy Expenditure. Cell Metab (2013) 17(4):534–48. doi: 10.1016/j.cmet.2013.03.005 PMC3646573 23562077

[96] Mano Y Shirabe K Yamashita Y Harimoto N Tsujita E Takeishi K. Preoperative Neutrophil-To-Lymphocyte Ratio Is a Predictor of Survival After Hepatectomy for Hepatocellular Carcinoma: A Retrospective Analysis. Ann Surg (2013) 258(2):301–5. doi: 10.1097/SLA.0b013e318297ad6b 23774313

[97] Malik HZ Prasad KR Halazun KJ Aldoori A Al-Mukhtar A Gomez D. Preoperative Prognostic Score for Predicting Survival After Hepatic Resection for Colorectal Liver Metastases. Ann Surg (2007) 246(5):806–14. doi: 10.1097/SLA.0b013e318142d964 17968173

[98] Tohme S Yazdani HO Al-Khafaji AB Chidi AP Loughran P Mowen K. Neutrophil Extracellular Traps Promote the Development and Progression of Liver Metastases After Surgical Stress. Cancer Res (2016) 76(6):1367–80. doi: 10.1158/0008-5472.can-15-1591 PMC4794393 26759232

[99] Mader S Pantel K. Liquid Biopsy: Current Status and Future Perspectives. Oncol Res Treat (2017) 40(7-8):404–8. doi: 10.1159/000478018 28693023

[100] Choudhury AD Werner L Francini E Wei XX Ha G Freeman SS. Tumor Fraction in Cell-Free DNA as a Biomarker in Prostate Cancer. JCI Insight (2018) 3(21):e122109. doi: 10.1172/jci.insight.122109 PMC6238737 30385733

[101] Phallen J Sausen M Adleff V Leal A Hruban C White J. Direct Detection of Early-Stage Cancers Using Circulating Tumor DNA. Sci Transl Med (2017) 9(403):eaan2415. doi: 10.1126/scitranslmed.aan2415 28814544 PMC6714979

[102] Alimirzaie S Bagherzadeh M Akbari MR. Liquid Biopsy in Breast Cancer: A Comprehensive Review. Clin Genet (2019) 95(6):643–60. doi: 10.1111/cge.13514 30671931

[103] Zhang Y Hu Y Ma C Sun H Wei X Li M. Diagnostic, Therapeutic Predictive, and Prognostic Value of Neutrophil Extracellular Traps in Patients With Gastric Adenocarcinoma. Front Oncol (2020) 10:1036. doi: 10.3389/fonc.2020.01036 32714865 PMC7344202

[104] Rivera-Franco MM Leon-Rodriguez E Torres-Ruiz JJ Gomez-Martin D Angles-Cano E de la Luz Sevilla-Gonzalez M. Neutrophil Extracellular Traps Associate With Clinical Stages in Breast Cancer. Pathol Oncol Res (2020) 26(3):1781–5. doi: 10.1007/s12253-019-00763-5 31656990

[105] Blasco A Coronado MJ Hernandez-Terciado F Martin P Royuela A Ramil E. Assessment of Neutrophil Extracellular Traps in Coronary Thrombus of a Case Series of Patients With Covid-19 and Myocardial Infarction. JAMA Cardiol (2020) 6(4):1–6. doi: 10.1001/jamacardio.2020.7308 PMC7772744 33372956

[106] Cahilog Z Zhao H Wu L Alam A Eguchi S Weng H. The Role of Neutrophil Netosis in Organ Injury: Novel Inflammatory Cell Death Mechanisms. Inflammation (2020) 43(6):2021–32. doi: 10.1007/s10753-020-01294-x PMC7443373 32830308

[107] Yang D Liu J. Neutrophil Extracellular Traps: A New Player in Cancer Metastasis and Therapeutic Target. J Exp Clin Cancer Res (2021) 40(1):233. doi: 10.1186/s13046-021-02013-6 34271947 PMC8283906

[108] Causey CP Jones JE Slack JL Kamei D Jones LE Subramanian V. The Development of N-A-(2-Carboxyl)Benzoyl-N(5)-(2-Fluoro-1-Iminoethyl)-L-Ornithine Amide (O-F-Amidine) and N-A;-(2-Carboxyl)Benzoyl-N(5)-(2-Chloro-1-Iminoethyl)-L-Ornithine Amide (O-Cl-Amidine) as Second Generation Protein Arginine Deiminase (Pad) Inhibitors. J Med Chem (2011) 54(19):6919–35. doi: 10.1021/jm2008985 PMC3196593 21882827

[109] Lewis HD Liddle J Coote JE Atkinson SJ Barker MD Bax BD. Inhibition of Pad4 Activity Is Sufficient to Disrupt Mouse and Human Net Formation. Nat Chem Biol (2015) 11(3):189–91. doi: 10.1038/nchembio.1735 PMC4397581 25622091

[110] Li M Lin C Deng H Strnad J Bernabei L Vogl DT. A Novel Peptidylarginine Deiminase 4 (Pad4) Inhibitor Bms-P5 Blocks Formation of Neutrophil Extracellular Traps and Delays Progression of Multiple Myeloma. Mol Cancer Ther (2020) 19(7):1530–8. doi: 10.1158/1535-7163.MCT-19-1020 PMC7335350 32371579

[111] Zeng J Xu H Fan PZ Xie J He J Yu J. Kaempferol Blocks Neutrophil Extracellular Traps Formation and Reduces Tumour Metastasis by Inhibiting Ros-Pad4 Pathway. J Cell Mol Med (2020) 24(13):7590–9. doi: 10.1111/jcmm.15394 PMC7339206 32427405

[112] Khan MA D'Ovidio A Tran H Palaniyar N. Anthracyclines Suppress Both Nadph Oxidase- Dependent and -Independent Netosis in Human Neutrophils. Cancers (Basel) (2019) 11(9):1328. doi: 10.3390/cancers11091328 PMC6770146 31500300

[113] Basyreva LY Voinova EV Gusev AA Mikhalchik EV Kuskov AN Goryachaya AV. Fluorouracil Neutrophil Extracellular Traps Formation Inhibited by Polymer Nanoparticle Shielding. Mater Sci Eng C Mater Biol Appl (2020) 108:110382. doi: 10.1016/j.msec.2019.110382 31924010

[114] Xia Y He J Zhang H Wang H Tetz G Maguire CA. Aav-Mediated Gene Transfer of Dnase I in the Liver of Mice With Colorectal Cancer Reduces Liver Metastasis and Restores Local Innate and Adaptive Immune Response. Mol Oncol (2020) 14(11):2920–35. doi: 10.1002/1878-0261.12787 PMC7607180 32813937

[115] Park HH Park W Lee YY Kim H Seo HS Choi DW. Bioinspired Dnase-I-Coated Melanin-Like Nanospheres for Modulation of Infection-Associated Netosis Dysregulation. Adv Sci (Weinh) (2020) 7(23–:2001940. doi: 10.1002/advs.202001940 33173718 PMC7645930

[116] Kajioka H Kagawa S Ito A Yoshimoto M Sakamoto S Kikuchi S. Targeting Neutrophil Extracellular Traps With Thrombomodulin Prevents Pancreatic Cancer Metastasis. Cancer Lett (2021) 497:1–13. doi: 10.1016/j.canlet.2020.10.015 33065249

[117] Shrestha B Ito T Kakuuchi M Totoki T Nagasato T Yamamoto M. Recombinant Thrombomodulin Suppresses Histone-Induced Neutrophil Extracellular Trap Formation. Front Immunol (2019) 10:2535. doi: 10.3389/fimmu.2019.02535 31736962 PMC6828967

[118] Rayes RF Vourtzoumis P Bou Rjeily M Seth R Bourdeau F Giannias B. Neutrophil Extracellular Trap-Associated Ceacam1 as a Putative Therapeutic Target to Prevent Metastatic Progression of Colon Carcinoma. J Immunol (2020) 204(8):2285–94. doi: 10.4049/jimmunol.1900240 PMC7534954 32169849

[119] Knuckley B Causey CP Jones JE Bhatia M Dreyton CJ Osborne TC. Substrate Specificity and Kinetic Studies of Pads 1, 3, and 4 Identify Potent and Selective Inhibitors of Protein Arginine Deiminase 3. Biochemistry (2010) 49(23):4852–63. doi: 10.1021/bi100363t PMC2884139 20469888

[120] Martins-Teixeira MB Carvalho I. Antitumour Anthracyclines: Progress and Perspectives. ChemMedChem (2020) 15(11):933–48. doi: 10.1002/cmdc.202000131 32314528

[121] Cao TM King MR. Supercharged Egfp-Trail Decorated Nets to Ensnare and Kill Disseminated Tumor Cells. Cell Mol Bioeng (2020) 13(4):359–67. doi: 10.1007/s12195-020-00639-8 PMC7479081 32952735

[122] Zhang Y Chandra V Riquelme Sanchez E Dutta P Quesada PR Rakoski A. Interleukin-17-Induced Neutrophil Extracellular Traps Mediate Resistance to Checkpoint Blockade in Pancreatic Cancer. J Exp Med (2020) 217(12):e20190354. doi: 10.1084/jem.20190354 32860704 PMC7953739

[123] Rivera-Franco MM Leon-Rodriguez E Sevilla-Gonzalez ML. Could Neutrophil Extracellular Traps Be the New Prognostic Markers of Cancer? Rev Invest Clin (2019) 71(6):365–8. doi: 10.24875/RIC.19003155 31823971

[124] Decker AS Pylaeva E Brenzel A Spyra I Droege F Hussain T. Prognostic Role of Blood Netosis in the Progression of Head and Neck Cancer. Cells (2019) 8(9):946. doi: 10.3390/cells8090946 PMC6770876 31438586

[125] Kaltenmeier CT Yazdani H van der Windt D Molinari M Geller D Tsung A. Neutrophil Extracellular Traps as a Novel Biomarker to Predict Recurrence-Free and Overall Survival in Patients With Primary Hepatic Malignancies. HPB (Oxford) (2021) 23(2):309–20. doi: 10.1016/j.hpb.2020.06.012 PMC7958967 32811764

[126] Zhang H Lv H Weng M Wang H Cata JP Chen W. Preoperative Leukocytosis Is Associated With Increased Tumor-Infiltrating Neutrophil Extracellular Traps and Worse Outcomes in Esophageal Cancer. Ann Transl Med (2020) 8(7):441. doi: 10.21037/atm.2020.03.190 32395485 PMC7210211

[127] Muqaku B Pils D Mader JC Aust S Mangold A Muqaku L. Neutrophil Extracellular Trap Formation Correlates With Favorable Overall Survival in High Grade Ovarian Cancer. Cancers (Basel) (2020) 12(2):505. doi: 10.3390/cancers12020505 PMC7072166 32098278

[128] Hamza MS Mousa SA. Cancer-Associated Thrombosis: Risk Factors, Molecular Mechanisms, Future Management. Clin Appl Thromb Hemost (2020) 26:1076029620954282. doi: 10.1177/1076029620954282 32877229 PMC7476343

[129] Thålin C Hisada Y Lundström S Mackman N Wallén H. Neutrophil Extracellular Traps: Villains and Targets in Arterial, Venous, and Cancer-Associated Thrombosis. Arterioscler Thromb Vasc Biol (2019) 39(9):1724–38. doi: 10.1161/atvbaha.119.312463 PMC6703916 31315434

